# In Silico-Based Design of a Hybrid Peptide with Antimicrobial Activity against Multidrug-Resistant *Pseudomonas aeruginosa* Using a Spider Toxin Peptide

**DOI:** 10.3390/toxins15120668

**Published:** 2023-11-23

**Authors:** Min Kyoung Shin, Hye-Ran Park, In-Wook Hwang, Kyung-Bin Bu, Bo-Young Jang, Seung-Ho Lee, Jin Wook Oh, Jung Sun Yoo, Jung-Suk Sung

**Affiliations:** 1 Department of Life Science, Dongguk University-Seoul, Goyang 10326, Republic of Korea; shinmk94@dgu.ac.kr (M.K.S.); 2019111678@dongguk.edu (H.-R.P.); hiw910@dongguk.edu (I.-W.H.); rudqls1211@dongguk.edu (K.-B.B.); by200015@dongguk.edu (B.-Y.J.); q969@dongguk.edu (S.-H.L.); oh5929@dongguk.edu (J.W.O.); 2 Species Diversity Research Division, National Institute of Biological Resources, Incheon 22689, Republic of Korea; lycosidae@korea.kr

**Keywords:** in silico design, antimicrobial peptide, multidrug-resistant *Pseudomonas aeruginosa*, ATCUN motif, synergistic effect

## Abstract

The escalating prevalence of antibiotic-resistant bacteria poses an immediate and grave threat to public health. Antimicrobial peptides (AMPs) have gained significant attention as a promising alternative to conventional antibiotics. Animal venom comprises a diverse array of bioactive compounds, which can be a rich source for identifying new functional peptides. In this study, we identified a toxin peptide, Lycotoxin-Pa1a (Lytx-Pa1a), from the transcriptome of the *Pardosa astrigera* spider venom gland. To enhance its functional properties, we employed an in silico approach to design a novel hybrid peptide, KFH-Pa1a, by predicting antibacterial and cytotoxic functionalities and incorporating the amino-terminal Cu(II)- and Ni(II) (ATCUN)-binding motif. KFH-Pa1a demonstrated markedly superior antimicrobial efficacy against pathogens, including multidrug-resistant (MDR) *Pseudomonas aeruginosa*, compared to Lytx-Pa1a. Notably, KFH-Pa1a exerted several distinct mechanisms, including the disruption of the bacterial cytoplasmic membrane, the generation of intracellular ROS, and the cleavage and inhibition of bacterial DNA. Additionally, the hybrid peptide showed synergistic activity when combined with conventional antibiotics. Our research not only identified a novel toxin peptide from spider venom but demonstrated in silico-based design of hybrid AMP with strong antimicrobial activity that can contribute to combating MDR pathogens, broadening the utilization of biological resources by incorporating computational approaches.

## 1. Introduction

Since the discovery of penicillin by Alexander Fleming in 1928, antibiotics have served as a pivotal resource for human health and saved countless lives [1]. In addition to a detailed understanding of bacterial physiology, the discovery of specific mechanisms to suppress microorganisms, such as interfering with cell wall synthesis and DNA function, has enabled the efficient control of infectious diseases [2,3]. However, the misuse and abuse of antibiotics have resulted in the emergence of multidrug-resistant (MDR) bacteria, which now pose a significant threat to humanity [4,5]. Compounding the issue, fewer new antibiotics are reaching the market as investment in the pharmaceutical industry gradually declines [6]. Therefore, there is an urgent need not only to explore new approaches for treating MDR bacterial infection but also to identify novel antimicrobial agents to address this problem.

Animal venom is a unique combination of proteins, peptides, and small molecules that paralyze prey or protect against predators [7,8]. Among these components, toxin peptides exhibit a high level of specificity for various targets, including receptors, enzymes, and biological membranes [9,10,11]. As they directly affect biological functions, studies on their activities, molecular mechanisms of action, and potential therapeutic applications are being conducted [12]. As one of the most diverse taxa of venomous animals, spiders are known to have distinct venom composition and toxin peptides with a high sequence and structural diversity as a result of their long evolutionary history [13]. Spider venom is also a major source of antimicrobial peptides (AMPs), which are crucial not only for hunting by depolarizing the cell membranes of prey but also for regulating innate immunity [14,15]. The wide-ranging inhibitory effects of AMPs on bacteria, fungi, and parasites make them promising candidates for the development of next-generation antibiotics.

Despite their significance in scientific and industrial applications, investigating animal venoms, particularly those originating from spiders, is often challenging using conventional proteomic methods due to their relatively small quantity. However, recent advances in technology and the introduction of computational approaches have opened a new era in the study of biological resources and venomics research [16]. With the development of sequencing technology, genetic information from venom and gland tissue can be obtained in a high-throughput manner, allowing for a multi-omics approach to a venom analysis [17,18]. Toxin peptides can be distinguished from non-toxin peptides through homology and an expression pattern analysis [19,20]. Furthermore, various in silico methods incorporating artificial intelligence (AI)-based techniques enable the prediction of both the functional properties and targets of these venomous components [21,22].

Peptides exhibit various functions in biological systems due to their structural diversity and molecular flexibility [23,24,25]. For example, AMPs, also known as host defense peptides (HDPs), possess a high net charge and show amphipathicity, enabling them to interact with the biomembranes of a wide range of pathogens and exhibit antimicrobial properties. In addition, they are key players in innate immunity and cell regulation, as they can selectively target specific molecules and receptors [26,27,28]. The presence of a functional motif in the peptide sequence can lead to distinct physiological functions such as cell entry, attachment, and signal transduction [29,30,31]. Among known motifs, the amino-terminal Cu(II)- and Ni(II)-binding (ATCUN) motif, which is found at the N-terminal of various naturally occurring proteins, induces DNA cleavage and the generation of reactive oxygen species (ROS) [32,33]. By incorporating such functional motifs, along with a computational analysis and prediction of structural and functional properties, it becomes possible to enhance the functionality of peptides and design hybrid peptides for specific purposes.

In this study, the toxin peptide Lycotoxin-Pa1a (Lytx-Pa1a) was identified by analyzing the transcriptome of the *Pardosa astrigera* spider venom. To enhance its functionality, a hybrid peptide called KFH-Pa1a originated from Lytx-Pa1a was designed by introducing the ATCUN motif and predicting its antibacterial and cytotoxic effects. KFH-Pa1a showed stronger antimicrobial effects against various pathogens, including MDR-*Pseudomonas aeruginosa*, compared to Lytx-Pa1a. It was confirmed that the hybrid peptide could rapidly inhibit bacterial growth by disrupting the cytoplasmic membrane, generating intrabacterial ROS, and binding and cleaving DNA. As the peptide exhibited synergistic activity with conventional antibiotics, this study not only identified a novel AMP from spider venom but also demonstrated the improved utilization of toxin peptides through computational approaches.

## 2. Results

### 2.1. Identification of Toxin Peptide Lycotoxin-Pa1a from the Transcriptome of Pardosa astrigera Spider Venom Gland

**Figure 1:**
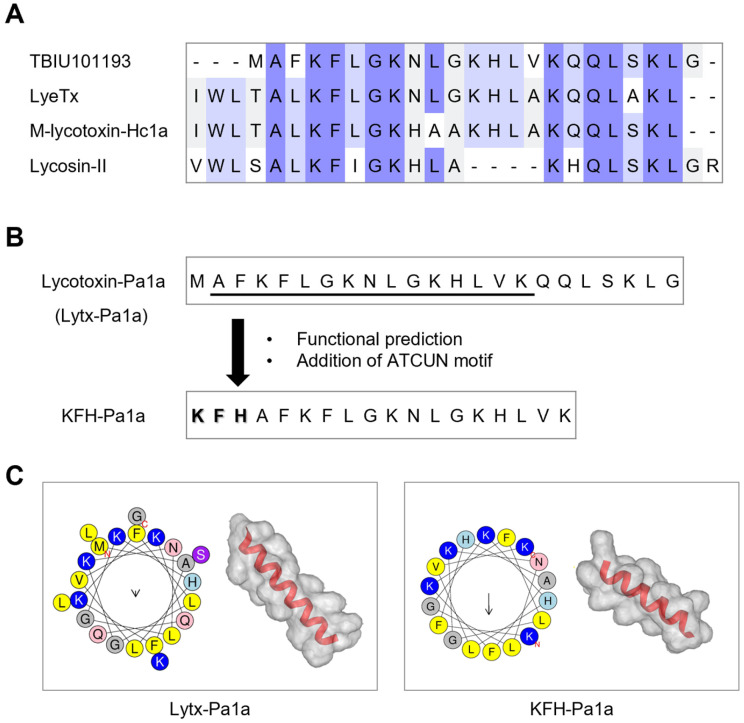
Identification of a spider toxin peptide, Lycotixn-Pa1a, and design of a hybrid peptide, KFH-Pa1a. (**A**) Homology analysis of the transcript TBIU101193 from the spider *Pardosa astrigera* venom gland transcriptome. The transcript showed significant sequence homology with known toxin peptides from wolf spider family and was named Lycotoxin-Pa1a (Lytx-Pa1a). (**B**) Schematic presentation of designing a hybrid peptide, KFH-Pa1a. In silico-based functional prediction (underlined region) and addition of ATCUN motif (KFH; bold letters) were utilized for the generation of KFH-Pa1a. (**C**) α-Helical property and three-dimensional structure of peptides were calculated using HeliQuest and PEP-FOLD4, respectively.

To identify a novel spider toxin peptide, the transcriptome of the *Pardosa astrigera* spider obtained from a previous study was analyzed [34]. The transcripts with >60% identity and/or >60% DB coverage were screened using the Basic Local Alignment Search Tool (BLAST) against the Uniprot database. As a result, TBIU101193 showed significant homology with known wolf-spider-derived toxin peptides, of which their antimicrobial effects were experimentally confirmed (Figure 1A). When compared with TBIU101193, LyeTx from *Lycosa erythrognatha*, M-lycotoxin-Hc1a from *Hogna carolinensis*, and Lycosin-II from *Lycosa singoriensis* each showed an identity score of 66.67%, 76.19%, and 68.42%, respectively. Also, the physiological and structural analysis revealed that TBIU101193 has a net charge of +5.1 and α-helical structure (Figure 1C and Table 1). As it was suggested as a spider toxin peptide that exhibits antimicrobial activity, the peptide sequence was named Lycotoxin-Pa1a (Lytx-Pa1a) and was further analyzed.

### 2.2. Design and Characterization of a Hybrid Peptide Based on Computational Approach

For the purpose of improving the utility of the identified toxin peptide, we sought to design a hybrid peptide with enhanced antimicrobial activity that could target both membranes and intrabacterial molecules by the introduction of the ATCUN motif. A series of in silico analyses were conducted according to a rational design strategy (Figure 1B). First, the sequence of Lytx-Pa1a was subjected to a sliding window of 15 mer sequences. ATCUN motifs reportedly found in humans were added for each generated sequence, followed by functional prediction using machine learning-based web tools for antimicrobial and hemolytic activity. The overall prediction results were used to select the most potent peptide. Following was the calculation of physiological properties of sequences, including molecular weight, net charge, water solubility, secondary structure, and α-helix propensity.

Finally, a hybrid peptide expected of improved functionality, KFH-Pa1a, was generated by adding the KFH motif from human histatin onto a partial sequence of Lytx-Pa1a. The prediction scores for antimicrobial activity rose from that of Lytx-Pa1a to KFH-Pa1a, while both peptides were predicted to have low hemolytic activity (Table 2). While there was no significant change in net charge, KFH-Pa1a gained a hydrophobic face among the helical structure (Table 1). Structural modeling predicted that KFH-Pa1a has an α-helical structure in an aqueous solution, similar to its template peptide. Based on in silico-based analyses, it was indicated that KFH-Pa1a would exhibit stronger inhibition against microbial activity than Lytx-Pa1a while not affecting human cells.

### 2.3. Anti-Microbial Activities of Lytx-Pa1a and KFH-Pa1a

**Figure 2:**
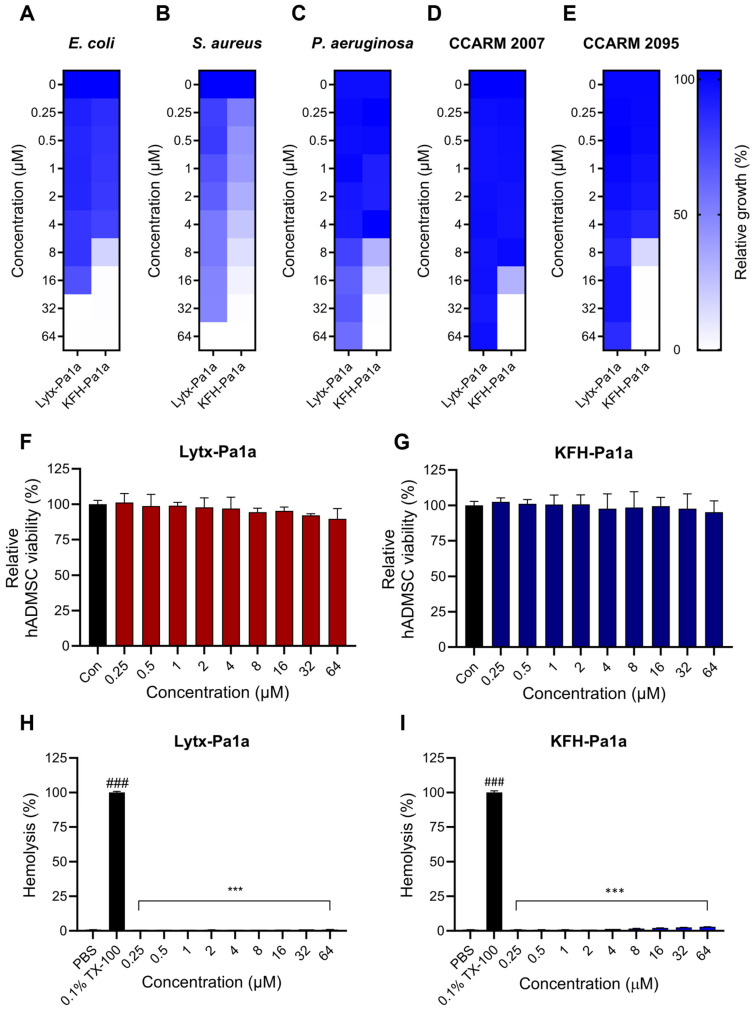
Evaluation of antibacterial activity and cytotoxicity of Lytx-Pa1a and KFH-PA1a. Peptide concentrations ranging from 0.25 to 64 μM were tested on pathogens, human ADMSCs, and bovine red blood cells (RBCs). Inhibitory effects of Lytx-Pa1a and KFH-Pa1a on (**A**) *Escherichia coli*, (**B**) *Staphylococcus aureus*, (**C**) *Pseudomonas aeruginosa*, (**D**) CCARM 2007, and (**E**) CCARM 2095 are shown. (**F**,**G**) Cell viability assay was performed on hADMSC to assess the cytotoxic effect of peptides. (**H**,**I**) Bovine RBCs were used to evaluate hemolytic activity of the peptides. Data are presented as mean ± SEM. *** *p* < 0.001 compared to the control. ### *p* < 0.001 compared between the negative control (PBS) and the positive control (0.1% Triton X-100; TX-100).

In order to determine the antibacterial activity of peptides, a minimum inhibitory concentration (MIC) assay was performed. The peptides were tested at concentrations ranging from 0.25 to 64 μM against pathogenic bacterial strains that can cause infectious disease. Lytx-Pa1a and KFH-Pa1a both showed completed inhibition of growth on the Gram-positive strain of *S*. *aureus* and Gram-negative strain of *E*. *coli* (Figure 2A,B). Interestingly, only KFH-Pa1a exhibited significant antibacterial activity against *P*. *aeruginosa* (Figure 2C). Therefore, it was tested whether the peptide can suppress the growth of MDR-*P*. *aeruginosa* (PA) isolates, CCARM 2007 and CCARM 2095, which have resistance to antibiotics groups of cephalosporin, ureidopenicillin, and quinolone. As a result, KFH-Pa1a inhibited CCARM 2007 and CCARM 2095, whereas Lytx-Pa1a showed no antibacterial activity even at the highest concentration (Figure 2D,E).

Next, the peptides were subjected to a cell viability assay and hemolysis assay to evaluate their cytotoxicity in normal animal cells. Primary human adipose-derived mesenchymal stem cells (hADMSCs) were treated with the same concentrations of peptides used in the MIC test. Lytx-Pa1a and KFH-Pa1a both had no significant effect on the cells up to 64 μM (Figure 2F,G). Bovine red blood cells (RBCs) were used to evaluate the hemolytic activity of the peptides. Additionally, 0.1% Triton X-100 (TX-100) was used as a positive control, which resulted in 100% hemolysis. In line with the cell viability assay, the results showed that the peptides have no significant hemolytic activity at all concentrations, with hemolytic activities under 5% (Figure 2H,I). The overall results indicated that Lytx-Pa1a and KFH-Pa1a are both AMPs with cytocompatibility, where the hybrid peptide showed more potent antimicrobial activity in accordance with its functional prediction.

### 2.4. Induction of Bacterial Membrane Disruption upon Lytx-Pa1a and KFH-Pa1a Treatment

**Figure 3:**
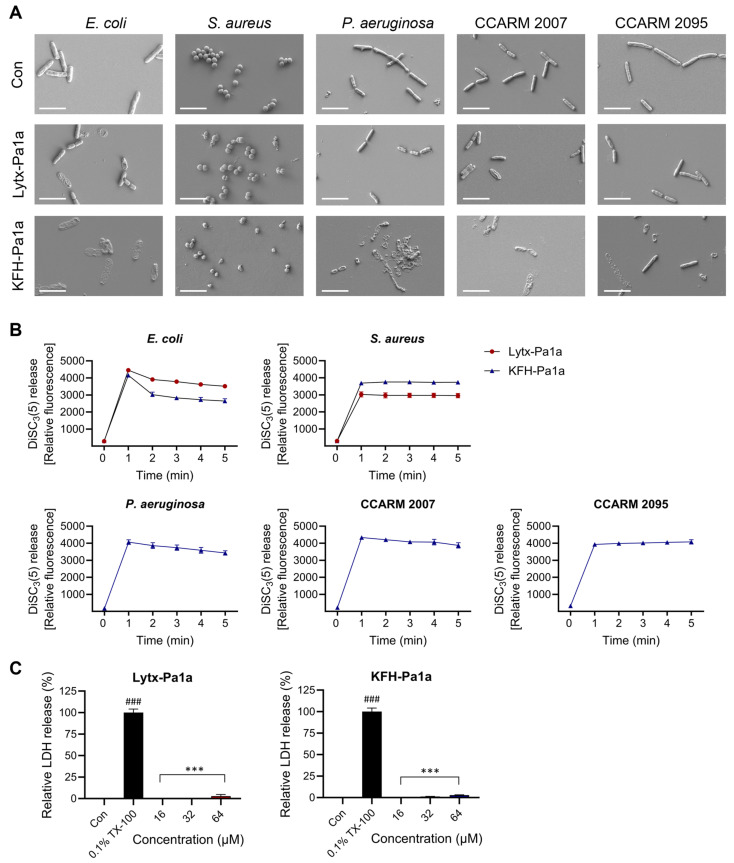
Effects of Lytx-Pa1a and KFH-Pa1a on bacterial and human cell membranes. (**A**) Field emission-scanning electron microscope imaging was performed to evaluate the effect of peptides on pathogens. Morphological changes of *E. coli* and *S. aureus* were observed by Lytx-Pa1a and KFH-Pa1a treatment. KFH-Pa1a induced disruption and lysis of bacterial cells of *P*. *aeruginosa*, CCARM 2007, and CCARM 2095. (**B**) Fluorescence dye DiSC3(5) was used to determine the depolarization of bacterial cytoplasmic membranes upon peptide treatment. A rapid increase in fluorescent signal was observed after treatment of Lytx-Pa1a and KFH-Pa1a. (**C**) Compared with the positive control (TX-100), both peptides exerted no significant lactate dehydrogenase release on hADMSC. Data are presented as mean ± SEM. ### *p* < 0.001 compared to the control. *** *p* < 0.001 compared between the positive control (TX-100) and the peptide-treated groups.

One of the major mechanisms by which AMPs exert antimicrobial activity is disrupting the integrity of bacterial membranes [35,36]. Thus, the morphological changes of pathogens were observed with field emission-scanning electron microscope (FE-SEM) imaging to find whether the peptides can interact with biomembranes of bacteria. All control groups remained with their normal shape, whereas treatment with 1× MIC Lytx-Pa1a and KFH-Pa1a for 2 h induced cell surface deformation and ruptures in *S*. *aureus* and *E*. *coli* (Figure 3A). In the KFH-Pa1a-treated group, *P*. *aeruginosa*, CCARM 2007, and CCARM 2095 cells were disrupted, and the small size of the observed bacteria indicated the rapid inhibition of growth and proliferation by the peptide.

The fluorescent dye 3,3′-Dipropylthiadicarbocyanine iodide, DiSC_3_(5), was used to assess the depolarization of bacterial membranes upon peptide treatment. Each strain was incubated with DiSC_3_(5) and then treated with 1× MIC, followed by immediate fluorescence signal detection. When compared with the PBS-treated control group, every peptide-treated group exhibited a gradual or sharp increase in the signal during 5 min observation (Figure 3B). The hybrid peptide was able to depolarize the cytoplasmic membrane of *P*. *aeruginosa* as well as MDR-PA. It was suggested that the KFH-Pa1a exhibited the general properties of AMPs, similar to the template peptide Lytx-Pa1a while having a stronger interaction with bacterial membranes.

Finally, a lactate dehydrogenase (LDH) release assay was performed to investigate the effect of peptides on human cell membranes. The three highest concentrations used in the previous assays (16, 32, 64 μM) were administered on hADMSCs for 24 h and were detected regarding extracellular LDH. When compared with the positive control, the 0.1%-TX-100-treated group, all of the peptide-treated groups showed almost no leakage of LDH (Figure 3C). It was confirmed that Lytx-Pa1a and KFH-Pa1a disrupt and permeabilize membranes specific to bacteria without affecting human cell membranes.

### 2.5. ROS Production and Bacterial DNA Cleavage by KFH-Pa1a

**Figure 4:**
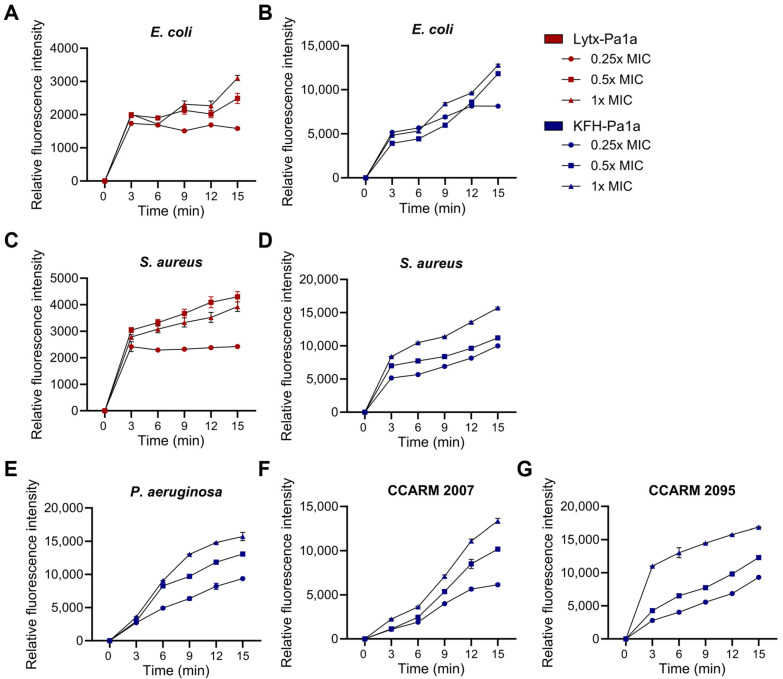
Intrabacterial ROS production upon Lytx-Pa1a and KFH-Pa1a treatment. Intrabacterial ROS levels were measured for 15 min immediately after treating 0.25×, 0.5×, and 1× MIC of Lytx-Pa1a or KFH-PA1a on (**A**,**B**) *E*. *coli*, (**C**,**D**) *S*. *aureus*, (**E**) *P. aeruginosa*, (**F**) CCARM 2007, and (**G**) CCARM 2095 strains.

As ATCUN motifs are known to induce oxidative damage by ROS production upon metal binding, 2′,7′-dichlorodihydrofluororesin diacetate (DCFH-DA) fluorescent dye was used to measure intrabacterial ROS levels after peptide treatment. It was shown that both peptides induced ROS production across all tested bacterial strains, where treatment of KFH-Pa1a was shown to generate significantly higher amounts of ROS compared with Lytx-Pa1a (Figure 4). This effect was not only time-dependent but also exhibited dose-dependent ROS generation at 0.25×, 0.5×, and 1× MIC concentrations. These results underscore the effectiveness of the ATCUN motif within KFH-Pa1a in promoting ROS production, which contributes to a crucial mechanism for bacterial inhibition.

**Figure 5:**
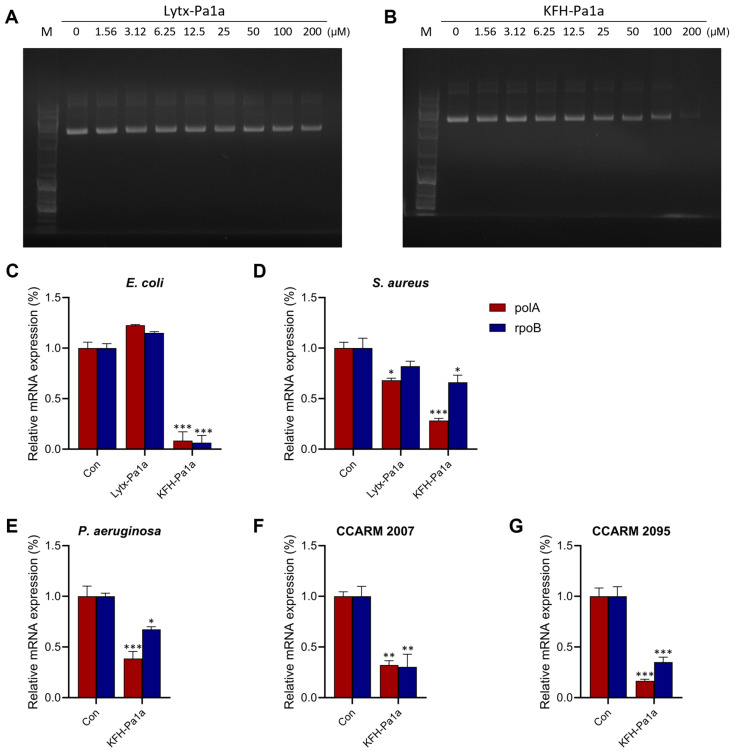
KFH-Pa1a induces DNA cleavage and inhibits DNA activity in bacteria. (**A**,**B**) Plasmid DNA samples were incubated with peptides for 15 min and subjected to agarose gel electrophoresis. KFH-Pa1a showed DNA cleavage activity in a dose-dependent manner. (**C**–**G**) Reverse transcription quantitative polymerase chain reaction revealed KFH-Pa1a significantly inhibits polA and rpoB activity in pathogens. Data are presented as mean ± SEM. * *p* < 0.01, ** *p* < 0.005, and *** *p* < 0.001 compared to the control.

We examined the DNA cleavage activity of peptides, which can result from the oxidative damage from the increased ROS level. Agarose gel electrophoresis was conducted after plasmid DNA was incubated with various concentrations of peptides for 15 min. In contrast to the peptide Lytx-Pa1a, which did not exhibit any DNA cleavage activity, KFH-Pa1a displayed a dose-dependent capacity for cleaving plasmid DNA (Figure 5A,B).

To further investigate the mechanisms underlying KFH-Pa1a’s ability to suppress bacterial DNA activity, we measured the mRNA expression levels of two essential bacterial genes, polA and rpoB, using the reverse transcription quantitative polymerase chain reaction (RT-qPCR) method. Remarkably, treatment with KFH-Pa1a resulted in a significant inhibition of gene expression for both polA and rpoB (Figure 5C–G). These findings suggest that KFH-Pa1a exerts its antibacterial effects, at least in part, through its capability to cleave DNA and suppress the transcription of crucial bacterial genes, highlighting its potential as a potent antimicrobial agent with DNA-targeting properties.

### 2.6. Synergistic Effect of KFH-Pa1a in Combination with Conventional Antibiotics

**Figure 6:**
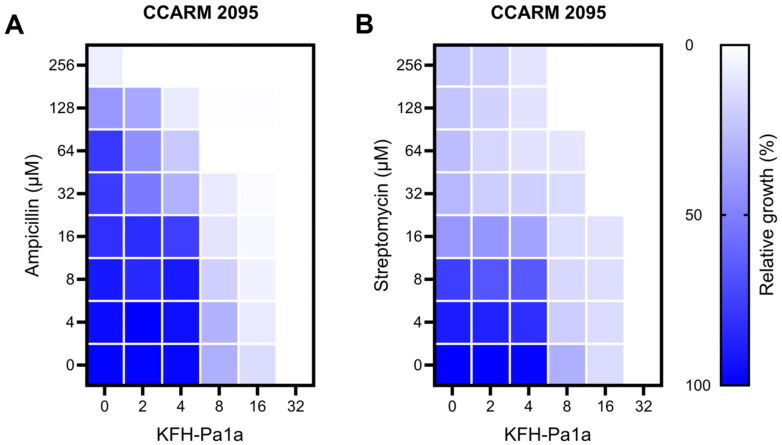
Checkerboard assay on MDR-PA CCARM 2095 using KFH-Pa1a and antibiotics. To investigate the synergistic effect of KFH-Pa1a and antibiotics, KFH-Pa1a (2–32 μM) was combined with conventional antibiotics (**A**) ampicillin and (**B**) streptomycin (4–256 μM) and administered on CCARM 2095.

The combinatorial activity of KFH-Pa1a and conventional antibiotics was measured with a checkerboard assay. CCARM 2095 is an MDR-PA strain that shows antibiotic resistance even at high concentrations of 256 uM of ampicillin and streptomycin. Based on the MIC of KFH-Pa1a, 32 μM, that was determined in the previous antimicrobial assay, a two-fold concentration of KFH-Pa1a ranging from 2 to 32 μM was cotreated onto CCARM 2095 with two-fold concentration of antibiotics ranging from 4 to 256 μM. The results demonstrated a considerable combinatorial effect when KFH-Pa1a was administered with antibiotics (Figure 6). This combined treatment exhibited significantly enhanced antibacterial activity compared to individual treatments with either KFH-Pa1a or antibiotics alone, where cotreatment of 16 μM of KFH-Pa1a and 32 μM of antibiotics was sufficient to inhibit the growth of CCARM 2095 completely. It was demonstrated that KFH-Pa1a exhibited a synergistic effect when combined with conventional antibiotics, showing potent antimicrobial activity when used both alone and together with antibiotics.

## 3. Discussion

The effectiveness of traditional antibiotics is progressively diminishing due to the emergence of antibiotic-resistant strains. Therefore, it is crucial to discover novel antibiotics and expand the range of clinically viable substances. In this study, we employed an in silico approach to identify a novel spider toxin from the venom gland transcripts of the *P*. *astrigera* spider and to design a hybrid AMP that exerts strong antibacterial activity against MDR-PA strains.

Animal venoms, due to their diverse composition and functional properties, represent a promising reservoir of bioactive molecules [37,38]. Among venomous organisms, spiders are renowned for harboring an extensive array of substances, including a spider-venom-derived peptide that exhibits various biological activities [39,40]. To uncover potential toxin peptides, we conducted a homology analysis using a *P*. *astrigera* venom gland transcript library. This analysis led to the identification of TBIU101193, named Lytx-Pa1a, which exhibited substantial homology to known spider toxin peptides. Also, the peptide structural and physiological analysis indicated that Lytx-Pa1a is a potential AMP having a high net charge and α-helical structure.

The application of computational approaches in the design of functional peptides represents a powerful and innovative strategy in the battle against infectious diseases. These approaches encompass a range of techniques, including a sequence and structural analysis, molecular modeling, and machine learning and deep learning algorithms for functional prediction. By harnessing these tools, researchers can predict the potential AMP candidates, optimize their sequences for improved specificity and efficacy, and even design novel peptides with desired functionalities. Based on the identified sequence of Lytx-Pa1a, a series of in silico techniques were employed to design a hybrid peptide expected to have stronger antimicrobial potential. Functional prediction was applied to obtain peptides with high antimicrobial activity while having low cytotoxicity. Additionally, the human ATCUN motif was introduced to generate multi-hit AMP, generating KFH-Pa1a.

The antimicrobial activity assay validated that KFH-Pa1a exhibited stronger inhibition on pathogens, especially suppressing the growth of MDR-PA strains. Also, the peptides had no significant effect on RBCs and hADMSCs. KFH-Pa1a showed general characteristics of AMPs similar to Lytx-Pa1a, interacting with bacterial membranes that led to depolarization of bacterial cells. In addition, KFH-Pa1a induced intrabacterial ROS generation and DNA damage, which may have contributed to enhanced antimicrobial activity. The introduction of the ATCUN motif in the design of antimicrobial peptides offers several notable advantages [41,42]. The ATCUN motif, characterized by its ability to bind transition metal ions, such as copper and nickel, imparts multifunctionality to the peptides. Upon binding with metal ions via four nitrogen molecules in the motif, a redox cycle by the copper–peptide complex can produce ROS, such as hydroxyl radicals. This enhances antimicrobial activity by facilitating the generation of oxidative stress within bacterial cells, leading to oxidative damage and cell death [33]. Secondly, the ATCUN motif can contribute to the selective targeting of bacterial membranes and molecules [43]. Furthermore, the ATCUN motif can augment the stability of the peptide structure and improve its overall pharmacokinetic properties [44]. Overall, incorporating the ATCUN motif represents a promising strategy for enhancing the antimicrobial efficacy and selectivity of peptide-based therapeutics, offering potential solutions to combat the rising challenge of antibiotic resistance in pathogenic bacteria.

Intrabacterial ROS production can effectively suppress and ultimately kill bacteria by inducing oxidative stress within the bacterial cell. ROS are highly reactive molecules that target cellular components like proteins, lipids, and DNA [45]. This oxidative damage disrupts vital cellular processes, including metabolism and DNA replication, leading to cellular dysfunction. ROS can also damage the bacterial cell membrane, causing leakage of ions and metabolites. Additionally, ROS can overwhelm the bacterial antioxidant defense systems, further compromising the bacterium’s ability to counteract oxidative stress. Thus, intrabacterial ROS production serves as a potent weapon against bacterial pathogens, contributing to their suppression and eventual demise [46,47].

Indeed, ROS measurement confirmed the dose-dependent production of ROS in pathogens by KFH-Pa1a. The following oxidative damage inside bacteria was determined with the DNA cleavage assay. Also, the peptide efficiently suppressed the bacterial DNA activity of polA and rpoB. PolA, or DNA Polymerase I, is a multifunctional enzyme in bacteria critical for DNA replication and repair. It synthesizes the complementary DNA strand during replication, removes RNA primers, and participates in DNA repair mechanisms. rpoB, RNA Polymerase Subunit Beta, is a vital component of RNA polymerase, responsible for bacterial transcription. It initiates transcription by binding to promoter regions, facilitates elongation of the RNA transcript, and contributes to transcription termination [48]. Together, PolA and rpoB play essential roles in DNA replication, transcription, and maintenance, ensuring the accurate transmission of genetic information, the adaptation to changing environments, and the overall survival of bacterial cells. These results demonstrate that the application of the ATCUN motif can be an effective solution to combat antimicrobial resistance.

By identifying and engineering the KFH-Pa1a hybrid peptide, we not only expanded the repertoire of spider-venom-derived AMPs but also demonstrated the substantial improvements achievable through computational approaches. KFH-Pa1a’s enhanced antimicrobial potency against drug-resistant pathogens underscores its potential as a valuable addition to the arsenal of antimicrobial agents. Furthermore, the observed synergistic effects with conventional antibiotics hold promise for combination therapies that can address the challenge of antibiotic resistance. These findings advance our capabilities in developing novel antimicrobial agents with the potential to combat drug-resistant infections effectively. Further research and clinical investigations are expected to explore the potential of KFH-Pa1a and the identification of functional peptides from biological resources in the fight against infectious diseases.

## 4. Materials and Methods

### 4.1. In Silico Analyses of Peptide Sequences

The multiple sequence alignment of toxin peptides was performed using UniProt BLAST (https://www.uniprot.org/blast (accessed on accessed on 5 June 2023)). Helical projection and three-dimensional modeling of peptides were conducted with HeliQuest (http://heliquest.ipmc.cnrs.fr/cgi-bin/ComputParams.py (accessed on 5 June 2023)) [49] and PEP-FOLD4 (https://bioserv.rpbs.univ-paris-diderot.fr/services/PEP-FOLD4 (accessed on 5 June 2023) [50], respectively. The following web tools were used for predicting antimicrobial and hemolytic activities of peptides: Database of Anti-Microbial peptides (ADAM; https://bioinformatics.cs.ntou.edu.tw/adam (accessed on 7 June 2023)) [51], AmpGram (http://biongram.biotech.uni.wroc.pl/AmpGram (accessed on 7 June 2023) [52], Database of Antimicrobial Activity and Structure of Peptides (DBAASP; https://dbaasp.org/tools?page=linear-amp-prediction (accessed on 7 June 2023) [53], CAMP_R3_ (http://www.camp3.bicnirrh.res.in (accessed on 7 June 2023) [54], and HemoPI (https://webs.iiitd.edu.in/raghava/hemopi (accessed on 7 June 2023) [55]. The physiological properties were calculated using the Innovagen protein calculator (https://pepcalc.com/protein-calculator.php (accessed on 5 June 2023) and HeliQuest.

### 4.2. Peptide Synthesis and Preparation

Peptides Lytx-Pa1a and KFH-Pa1a were synthesized using the solid phase method from Biostem (Ansan, Republic of Korea). The peptide purity was obtained at >95% and was verified with high-performance liquid chromatography and mass spectroscopy. The peptides were reconstituted to reach 1 mM in PBS containing 0.5 mM of CuCl_2_. The samples were aliquoted and stored at −80 °C before use.

### 4.3. Bacterial Strains and Cell Line

The following bacterial strains were used in the study: *Escherichia coli* KCCM 11234, *Staphylococcus aureus* KCCM 11335, *Pseudomonas aeruginosa* ATCC 9027, and MDR *P*. *aeruginosa* isolates CCARM 2007 and CCARM 2095. Bacterial cultures were grown in tryptic soy agar (TSA, Difco Laboratories, Detroit, MI, USA) at 37 °C. Adipose-derived mesenchymal stem cells (hADMSCs) were purchased from CEFO Co. (Seoul, Republic of Korea) and maintained in a CEFOgro™ Human MSC Growth Medium (CEFO Co.) at 37 °C under humidified air with 5% CO_2_.

### 4.4. Antimicrobial Activity Measurement

For the MIC measurement, the bacterial inoculant was diluted to 2 × 10^5^ CFU/mL, where 100 μL of the diluent was transferred into a 96-well plate and then mixed with an equal volume of peptides (0.25–64 μM). The samples were incubated at 37 °C overnight, and the absorbance was measured at 600 nm using a microplate reader (Molecular Devices, Sunnyvale, CA, USA). The MIC values were calculated as the lowest peptide concentration with no observable bacterial growth.

### 4.5. Cell Viability Assay

To determine the cytotoxicity of peptides, hADMSCs were seeded at a density of 1 × 10^4^ cells/well in 96-well plates. After 24 h, peptides ranging from 0.25 μM to 64 μM were administered onto cells and incubated for another 24 h. For each well, 10 μL of a Quanti-Max WST-8 Cell Viability assay solution (Biomax, Seoul, Republic of Korea) was added, and the absorbance was read at 450 nm using a microplate reader (Molecular Devices).

### 4.6. Hemolytic Activity

Bovine RBCs were purchased from Innovative Research (Novi, MI, USA) and diluted to a 4% (*v*/*v*) suspension in PBS. Each 100 μL of RBC aliquots was incubated with an equal amount of peptide samples, followed by incubation for 1 h at 37 °C. For controls, 0% and 100% hemolysis were determined by adding PBS and 0.1% Triton X-100 (TX-100) to RBC aliquots, respectively. The supernatants from samples were transferred to a 96-well microplate after centrifugation at 3000× *g* for 10 min at 4 °C. The absorbance (Ab) was measured and used for calculating hemolytic activity using the following equation:

$$Hemolytic\;activity\;\left(\%\right)=\frac{Ab_{TX-100}-Ab_{PBS}}{Ab_{peptide}-Ab_{PBS}}\times 100$$

### 4.7. FE-SEM Imaging

Bacterial cultures in the mid-log phase were seeded on poly-L-lysine (Sigma-Aldrich, St. Louis, MO, USA)-coated cover slides and incubated for 2 h. The samples were treated with peptides for another 2 h, followed by fixation using 2.5% glutaraldehyde (Sigma-Aldrich) overnight. The cover slides were dehydrated using 30% to 100% ethanol and coated with platinum. The morphological observation of pathogens was conducted using a Hitachi S-4300 FE-SEM (Tokyo, Japan).

### 4.8. Membrane Depolarization Measurement

Bacteria inoculants were cultured overnight and then diluted into 1 × 10^7^ CFU/mL in a 5 mM HEPES buffer. Diluents were washed three times and resuspended in the same buffer containing 0.4 μM of DiSC_3_(5). Resuspension of 100 μL was added in each well of a black 96-well microplate. After a 30 min incubation in the dark at 37 °C, the fluorescence was measured immediately after adding 2× peptide samples for 5 min (excitation: 622 nm; emission: 670 nm) using an Infinite F200 Pro multimode microplate reader (Tecan, Männedorf, Switzerland).

### 4.9. LDH Release Assay

ADMSCs were seeded on 96-well plates (1 × 10^4^ cells/well) and maintained for 24 h. The peptides were treated for another 24 h, followed by addition of 50 μL of Lactate Assay kit II (Sigma-Aldrich, St. Louis, MO, USA) in each well. After a 15 min incubation at room temperature, the absorbance was measured at 450 nm using a microplate reader (Molecular Devices, San Jose, CA, USA).

### 4.10. ROS Measurement

Bacterial cells were pelletized with centrifugation at 3300× *g*, 4 °C, for 7 min. After washing, the samples were resuspended in 10 μM of DCF-DA in PBS and incubated for 30 min at 37 °C in a shaking incubator. Bacterial resuspensions of 100 μL were transferred into a black 96-well microplate and then mixed with 100 μL of the peptide sample (0.25×, 0.5×, and 1× MIC). The fluorescent signal (excitation: 485 nm, emission: 535 nm) was read with the Infinite F200 Pro multimode microplate reader (Tecan) for 15 min.

### 4.11. Agarose Gel Electrophoresis

For each peptide concentration, 400 ng of pET28b plasmid DNA was mixed in a total of 20 μL and incubated for 15 min at room temperature. After adding a DNA sample buffer, each sample was separated with agarose gel electrophoresis using 1% agarose gel at 50 V for 45 min.

### 4.12. qRT-PCR

Bacterial cultures of 1 × 10^9^ CFU were incubated with peptides for 1 h under a shaking condition at 37 °C. Total RNA was isolated using a Trizol reagent (Sigma-Aldrich), and 2000 ng of RNA was used to synthesize cDNA with M-MLV reverse transcriptase (ELPISBIO, Daejeon, Republic of Korea). RT-qPCR was performed to amplify 16s, polA, and rpoB (Table 3) by using an SYBR Green PCR master mix (KAPA Biosystems, Wilmington, MA, USA).

### 4.13. Statistical Analysis

All experiments were conducted in triplicate and the results are expressed as the mean ± standard error of the mean (SEM). The statistical analyses were performed using a one-way ANOVA test followed by Tukey’s post-test on GraphPad Prism 9.3.1 (GraphPad Software, La Jolla, CA, USA). *p*-Values less than 0.05 were considered to indicate statistically significant differences.

## Figures and Tables

**Table 1:** Physicochemical characterization of Lytx-Pa1a and KFH-Pa1a.

Peptide	Length	Molecular Weight	Net Charge	Water Solubility	Hydrophobic Face
Lytx-Pa1a	23	2586.15	+5.1	Good	None
KFH-Pa1a	18	2112.56	+5.2	Good	LFLGF

**Table 2:** Functional prediction results of Lytx-Pa1a and KFH-Pa1a.

Peptide	Antimicrobial Activity				Hemolytic Activity	
	ADAM	AmpGram	DBAASP_V3_	CAMPR3 *	HemoPI	DBAASP
Lytx-Pa1a	1.86	1.000	AMP	0.816	0.50	Not Active
KFH-Pa1a	2.96	1.000	AMP	0.925	0.46	Not Active

* Support vector machine classifier.

**Table 3:** Primer sequences used for qRT-PCR.

Gene	Forward	Reverse
*E*. *coli* 16s	CACACTGGAACTGAGACACG	GCTTCTTCTGCGGGTAACG
*E*. *coli* polA	ATGGTTCAGATCCCCCAA	TTCTACGCCAGAAACCGCCA
*E*. *coli* rpoB	AGACCGTTTCACCACCATCC	TCGGCGTTACCTTACCAACC
*S*. *aureus* 16s	GTAGGTGGCAAGCGTTATCC	CGCACATCAGCGTCAG
*S*. *aureus* polA	TTCATTGGAAGAAGCGGCCA	AACCATGAAACTAGTTCGGCA
*S*. *aureus* rpoB	GAACATGCAACGTCAAGCAG	AATAGCCGCACCAGAATCAC
*P*. *aeruginosa* 16s	CAAAACTACTGAGCTAGAGTACG	TAAGATCTCAAGGATCCCAACGGGT
*P*. *aeruginosa* polA	TCAACACCATGACCGGTAGC	GGGGATGTTGTCGACCTTGT
*P*. *aeruginosa* rpoB	CTGATCATCTTCGACCGCGA	TTCTCGGTCATCAGGGGGAT

## Data Availability

The data of this study are available from the corresponding author (Jung-Suk Sung) upon reasonable request.
